# The paired immunoglobulin-like type 2 receptor alpha (PILRA) gene polymorphism rs1859788 reduces risk of Alzheimer’s Disease in men homozygous for the ApoE ε4 allele

**DOI:** 10.21203/rs.3.rs-4798019/v1

**Published:** 2024-07-29

**Authors:** Steven Lehrer, Peter Rheinstein

**Affiliations:** Icahn School of Medicine Mount Sinai; Severn Health Solutions

**Keywords:** dementia, PILRA, phewas, anemia

## Abstract

**Background:**

The APOE gene has long been associated with Alzheimer Disease (AD) risk. Emerging research indicates that other genetic loci, including the paired immunoglobulin-like type 2 receptor alpha (PILRA) gene, may play a crucial role. In the current study we used UK Biobank data to assess the relationship between PILRA and AD.

**Methods:**

We examined the PILRA polymorphism rs1859788, a single nucleotide missense variant, G > A, minor allele frequency 0.3. Single nucleotide polymorphism (SNP) data for rs429358 and rs7412 determined APOE isoform. We used PheWeb to perform a phenome wide association study (phewas) of rs1859788 and identify other conditions that might be related to both AD and rs1859788.

**Results:**

In male subjects homozygous for ApoE isoform ε4/ε4, of the men without AD, 9.7% had AA genotype; of the men with AD, 1.8% had AA genotype. This difference was significant (p = 0.006, two tail Fisher exact test). In female subjects homozygous for ApoE isoform ε4/ε4, of the women without AD, 10.4% had AA genotype; of the women with AD 7.9% had AA genotype. This difference was not significant (p = 0.481). In subjects not homozygous for ApoE isoform ε4/ε4, the effect of PILRA genotype was not significant. A phewas of rs1859788 found an association with megaloblastic anemia.

**Conclusion:**

We have confirmed the previously noted PILRA snp rs1859788 risk reduction of AD, as well as a PILRA link to the ApoE ε4 isoform that has been previously described. We are uncertain why the significant association is only with men who are homozygous for the ε4/ε4 isoform. A phewas indicated that PILRA SNP rs1859788 is associated with megaloblastic anemia, which may explain an observed association between AD and anemia. The identification of PILRA as a potential risk gene for Alzheimer’s disease underscores the complexity of the genetic landscape contributing to AD. Alongside APOE, PILRA may play a significant role in modulating key pathological processes such as neuroinflammation and amyloid-beta accumulation.

## Introduction

Alzheimer’s disease affects millions worldwide and poses a significant public health challenge. AD is characterized by progressive cognitive decline and neuropathological hallmarks, including amyloid-beta (Aβ) plaques and neurofibrillary tangles. AD is a complex disease involving genetic, environmental, and lifestyle factors. The APOE gene has long been associated with AD risk. Emerging research indicates that other genetic loci, including the paired immunoglobulin-like type 2 receptor alpha (PILRA) gene, may play a crucial role. Understanding these genetic influences is critical for developing targeted interventions and improving diagnostic accuracy.

The APOE gene, located on chromosome 19, encodes apolipoprotein E, a protein involved in lipid metabolism and neuronal repair. The ε4 allele of APOE significantly increases AD risk, while the ε2 allele is protective. PILRA, located on chromosome 7q21, encodes an inhibitory receptor expressed on myeloid cells. A common missense variant (G78R, rs1859788) of PILRA may be protective against AD [1].

In the current study we used UK Biobank data to assess the relationship between PILRA and AD.

## Methods

The UK Biobank is a large prospective observational study comprising approximately 500,000 men and women (N = 229,134 men, N = 273,402 women), more than 90% white, aged 40–69 years at enrollment. Participants were recruited from across 22 centers located throughout England, Wales, and Scotland between 2006 and 2010 and continue to be longitudinally followed for capture of subsequent health events [2]. UK Biobank has approval from the Northwest Multi-center Research Ethics Committee (MREC) to obtain and disseminate data and samples from the participants, and these ethical regulations cover the work in this study. Written informed consent was obtained from all participants. Details can be found at www.ukbiobank.ac.uk/ethics. Our UK Biobank application was approved as UKB project 57245 (S.L., P.H.R.). Our analysis included all subjects genotyped for PILRA in the UK Biobank database.

We examined the PILRA single nucleotide polymorphism (SNP) rs1859788, a single nucleotide missense variant, G > A, minor allele frequency 0.3.

SNP data for rs429358 and rs7412 determined APOE isoform [3]. We used PheWeb [4] to perform a phenome wide association study (phewas) of rs1859788 to identify other conditions that might be related to both AD and rs1859788.

Data processing was performed on Minerva, a Linux mainframe with Centos 7.6, at the Icahn School of Medicine at Mount Sinai. We used PLINK, a whole-genome association analysis toolset, to analyze the UKB chromosome files [5]. Statistical analysis was done with SPSS 26.

## Results

Table 1 displays demographics of 483,936 subjects in the study.

Table 2 displays analysis of 9754 subjects homozygous for ApoE isoform ε4/ε4, PILRA genotype (GG, AG, AA) versus AD (yes or no). Of the men without AD, 9.7% had AA genotype; of the men with AD, 1.8% had AA genotype. This difference was significant (p = 0.006, two tail Fisher exact test). Of the women without AD, 10.4% had AA genotype; of the women with AD 7.9% had AA genotype. This difference was not significant (p = 0.481). In subjects not homozygous for ApoE isoform ε4/ε4, the effect of PILRA genotype was not significant.

**Figure 1:**
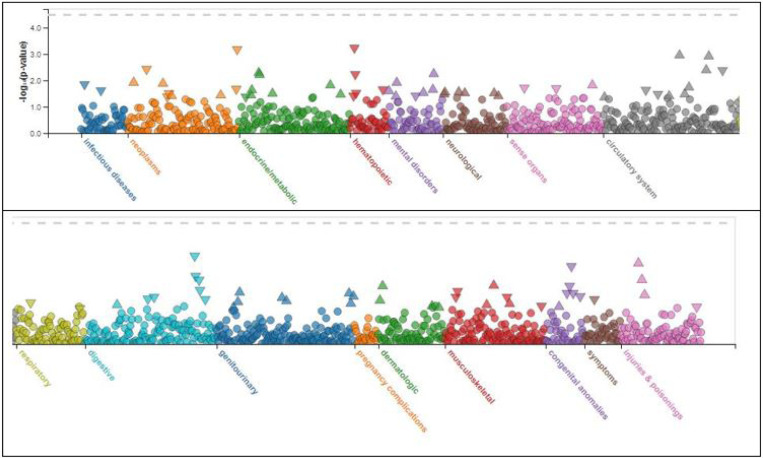
Phewas of rs1859788.

A phewas of rs1859788 found an association with megaloblastic anemia (figure 1 and table 3).

## Discussion

We have confirmed the previously noted PILRA SNP rs1859788 risk reduction of AD, as well as a PILRA link to the ApoE ε4 isoform that has been previously described [6]. We are uncertain why the significant association is only with men who are homozygous for the ε4/ε4 isoform.

A phewas (figure 1 and table 3) indicated that PILRA SNP rs1859788 is associated with megaloblastic anemia, which may explain an observed association between AD and anemia. In one study, individuals with anemia had a 41% increased risk of developing AD and a 34% increased risk of developing any kind of dementia compared to those without anemia [7]. Another study found that people with AD were often anemic, though not iron deficient [8]. However, pernicious anemia can present with confusion, difficulty concentrating, memory loss, cognitive decline, and be mistaken for AD [9].

PILRA is a member of the paired immunoglobulin-like type 2 receptor family and plays a role in immune regulation. It is an inhibitory receptor that modulates immune responses by interacting with its ligands on the surface of other cells. Whole exome sequencing studies (WES) have identified PILRA as a potential risk gene for AD, although its exact mechanism in disease pathology remains to be fully elucidated [10].

Recent studies suggest that PILRA may influence neuroinflammation, a key feature of AD. Neuroinflammation is driven by the activation of glial cells, which release inflammatory cytokines that can exacerbate neuronal damage. PILRA may affect the regulation of these immune responses, potentially modulating the inflammatory environment in the AD brain.

People who have PILRA gene loss-of-function mutations such as rs1859788 are protected from AD. Microglia increase their metabolism and reduce inflammatory reactions in the absence of PILRA, thereby ameliorating AD. A PILRA antibody replicates these actions and may be therapeutic for AD [11].

The interplay between APOE and PILRA in AD is an emerging area of research. Both genes are involved in processes that are central to AD pathology, such as amyloid-beta metabolism and neuroinflammation. PILRA may interact with APOE to influence the inflammatory response to amyloid-beta accumulation. PILRA could modulate the activation of glial cells in APOE ε4 carriers, affecting the severity of inflammation and subsequent neuronal damage.

Understanding the genetic interactions between PILRA and APOE may provide insights into the heterogeneity of AD and help identify novel therapeutic targets. Personalized medicine approaches could consider these genetic factors to develop targeted interventions aimed at modifying disease progression in individuals with specific genetic profiles.

## Conclusion

The identification of PILRA as a potential risk gene for Alzheimer’s disease underscores the complexity of the genetic landscape contributing to AD. Alongside APOE, PILRA may play a significant role in modulating key pathological processes such as neuroinflammation and amyloid-beta accumulation. Further research into the interactions between these genes and their contributions to AD pathophysiology will be crucial for developing new therapeutic strategies and improving our understanding of this devastating disease.

## Figures and Tables

**Table 1:** Demographics of 483936 subjects included in the study.

	control	AD
N	482983	953
age	56 ± 8.1	64 ± 4
sex	57% female	54% female
years education	15 ± 5	12 ± 5
race	95% white	95% white

**Table 2:** Analysis of 9754 subjects homozygous APOE ε4/ε4, PILRA genotype (GG, AG, AA) versus AD (yes or no). Of the men without AD, 9.7% had AA genotype; of the men with AD, 1.8% had AA genotype. This difference was significant (p = 0.006, two tail Fisher exact test). Of the women without AD, 10.4% had AA genotype; of the women with AD 7.9% had AA genotype. This difference was not significant (p = 0.481).

				AD	AD		
				no	yes		p value
women	genotype	GG	Count	2518	27	2545	0.481
			% within AD	48.00%	42.90%	47.90%	
		AG	Count	2184	31	2215	
			% within AD	41.60%	49.20%	41.70%	
		AA	Count	546	5	551	
			% within AD	10.40%	7.90%	10.40%	
	Total		Count	5248	63	5311	
			% within AD	100.00%	100.00%	100.00%	
men	genotype	GG	Count	2047	37	2084	0.006
			% within AD	46.70%	66.10%	46.90%	
		AG	Count	1914	18	1932	
			% within AD	43.60%	32.10%	43.50%	
		AA	Count	426	1	427	
			% within AD	9.70%	1.80%	9.60%	
	Total		Count	4387	56	4443	
			% within AD	100.00%	100.00%	100.00%	
Total	genotype	GG	Count	4565	64	4629	0.123
			% within AD	47.40%	53.80%	47.50%	
		AG	Count	4098	49	4147	
			% within AD	42.50%	41.20%	42.50%	
		AA	Count	972	6	978	
			% within AD	10.10%	5.00%	10.00%	
	Total		Count	9635	119	9754	
			% within AD	100.00%	100.00%	100.00%	

**Table 3:** Phewas association results.

Category	Phenotype	P-value	Effect Size (se)	Number of samples (cases/controls)
digestive	Cholelithiasis and cholecystitis	5.30E-04	−0.10 (0.029)	2939/ 47150
hematopoietic	Megaloblastic anemia	5.80E-04	−0.43 (0.12)	155 / 38182
neoplasms	Benign neoplasm of lymph nodes	6.60E-04	−0.14 (0.043)	1410 / 47332
injuries & poisonings	Skull and face fracture and other intercranial injury	1.00E-03	0.15 (0.046)	1160 / 49171
circulatory system	Symptoms involving cardiovascular system	1.10E-03	0.15 (0.046)	1191 / 45087
circulatory system	Peripheral vascular disease, unspecified	1.20E-03	0.12 (0.036)	1978 / 43682
congenital anomalies	Congenital musculoskeletal deformities of spine	1.30E-03	−0.18 (0.056)	778 / 49387
digestive	Cholelithiasis	3.00E-03	−0.094 (0.032)	2509 / 47150
neoplasms	Malignant neoplasm of gallbladder and extrahepatic bile ducts	3.70E-03	−0.43 (0.15)	112 / 42041
circulatory system	Peripheral vascular disease	4.00E-03	0.090 (0.031)	2700 / 43682

## References

[1] Rathore N, Ramani SR, Pantua H, Payandeh J, Bhangale T, Wuster A, Kapoor M, Sun Y, Kapadia SB, Gonzalez L, Zarrin AA, Goate A, Hansen DV, Behrens TW, Graham RR (2018) Paired Immunoglobulin-like Type 2 Receptor Alpha G78R variant alters ligand binding and confers protection to Alzheimer’s disease. PLoS Genet 14:e1007427 30388101 10.1371/journal.pgen.1007427 PMC6235402

[2] Arthur RS, Wang T, Xue X, Kamensky V, Rohan TE (2020) Genetic Factors, Adherence to Healthy Lifestyle Behavior, and Risk of Invasive Breast Cancer Among Women in the UK Biobank. J Natl Cancer Inst 112:893–901 31899501 10.1093/jnci/djz241 PMC7492765

[3] Kuo CL, Pilling LC, Atkins JL, Masoli JAH, Delgado J, Kuchel GA, Melzer D (2020) APOE e4 genotype predicts severe COVID-19 in the UK Biobank community cohort. J Gerontol A Biol Sci Med Sci Published online 2020 May 26. 10.1093/gerona/glaa131 PMC7314139 32451547

[4] Gagliano Taliun SA, VandeHaar P, Boughton AP, Welch RP, Taliun D, Schmidt EM, Zhou W, Nielsen JB, Willer CJ, Lee S (2020) Exploring and visualizing large-scale genetic associations by using PheWeb. Nat Genet 52:550–552 32504056 10.1038/s41588-020-0622-5 PMC7754083

[5] Chang CC, Chow CC, Tellier LC, Vattikuti S, Purcell SM, Lee JJ (2015) Second-generation PLINK: rising to the challenge of larger and richer datasets. Gigascience 4:7 25722852 10.1186/s13742-015-0047-8 PMC4342193

[6] Lopatko Lindman K, Jonsson C, Weidung B, Olsson J, Pandey JP, Prokopenko D, Tanzi RE, Hallmans G, Eriksson S, Elgh F, Lovheim H (2022) PILRA polymorphism modifies the effect of APOE4 and GM17 on Alzheimer’s disease risk. Sci Rep 12:13264 35918447 10.1038/s41598-022-17058-6 PMC9346002

[7] Wolters FJ, Zonneveld HI, Licher S, Cremers LGM, Heart Brain Connection Collaborative, Research G, Ikram MK, Koudstaal PJ, Vernooij MW, Ikram MA (2019) Hemoglobin and anemia in relation to dementia risk and accompanying changes on brain MRI. Neurology 93:e917–e926 31366722 10.1212/WNL.0000000000008003 PMC6745727

[8] Hare DJ, Doecke JD, Faux NG, Rembach A, Volitakis I, Fowler CJ, Grimm R, Doble PA, Cherny RA, Masters CL, Bush AI, Roberts BR (2015) Decreased plasma iron in Alzheimer’s disease is due to transferrin desaturation. ACS Chem Neurosci 6:398–402 25588002 10.1021/cn5003557

[9] Htut TW, Thein KZ, Oo TH (2021) Pernicious anemia: Pathophysiology and diagnostic difficulties. J Evid Based Med 14:161–169 34015185 10.1111/jebm.12435

[10] Patel T, Brookes KJ, Turton J, Chaudhury S, Guetta-Baranes T, Guerreiro R, Bras J, Hernandez D, Singleton A, Francis PT (2018) Whole‐exome sequencing of the BDR cohort: evidence to support the role of the PILRA gene in Alzheimer’s disease. Neuropathol Appl Neurobiol 44:506–521 29181857 10.1111/nan.12452 PMC6005734

[11] Monroe K, Weerakkody T, Sabelström H, Tatarakis D, Suh J, Chin M, Andrews S, Propson N, Balasundar S, Davis S (2024) PILRA regulates microglial neuroinflammation and lipid metabolism as a candidate therapeutic target for Alzheimer’s disease. Res Square. 10.21203/rs.3.rs-3954863/v1,15 Feb, 2024

